# Heterogeneity of Calcium Responses to Secretagogues in Corticotrophs From Male Rats

**DOI:** 10.1210/en.2017-00107

**Published:** 2017-03-16

**Authors:** Nicola Romanò, Heather McClafferty, Jamie J. Walker, Paul Le Tissier, Michael J. Shipston

**Affiliations:** 1 Centre for Integrative Physiology, University of Edinburgh, Edinburgh EH8 9XD, United Kingdom; ^2^Centre for Biomedical Modelling and Analysis, University of Exeter, Exeter EX2 5DW, United Kingdom; 3 Engineering and Physical Sciences Research Council Centre for Predictive Modelling in Healthcare, University of Exeter, Exeter EX4 4QJ, United Kingdom; 4 College of Engineering, Mathematics and Physical Sciences, University of Exeter, Exeter EX4 4QF, United Kingdom; 5 Henry Wellcome Laboratories for Integrative Neuroscience and Endocrinology, University of Bristol, Bristol BS1 3NY, United Kingdom

## Abstract

Heterogeneity in homotypic cellular responses is an important feature of many biological systems, and it has been shown to be prominent in most anterior pituitary hormonal cell types. In this study, we analyze heterogeneity in the responses to hypothalamic secretagogues in the corticotroph cell population of adult male rats. Using the genetically encoded calcium indicator GCaMP6s, we determined the intracellular calcium responses of these cells to corticotropin-releasing hormone and arginine-vasopressin. Our experiments revealed marked population heterogeneity in the response to these peptides, in terms of amplitude and dynamics of the responses, as well as the sensitivity to different concentrations and duration of stimuli. However, repeated stimuli to the same cell produced remarkably consistent responses, indicating that these are deterministic on a cell-by-cell level. We also describe similar heterogeneity in the sensitivity of cells to inhibition by corticosterone. In summary, our results highlight a large degree of heterogeneity in the cellular mechanisms that govern corticotroph responses to their physiological stimuli; this could provide a mechanism to extend the dynamic range of the responses at the population level to allow adaptation to different physiological challenges.

## Teaser

We show prominent heterogeneity in several aspects of the calcium responses to hypothalamic secretagogues in corticotrophs of adult male rats.

Variability in homotypic cell function is a ubiquitous feature in biology, appearing in a wide range of situations, such as gene expression (1, 2), gene networks (3), molecular pathways (4, 5), and cell behavior (6). This “biological noise” can result from complex nondeterministic molecular dynamics at the microscopic level, and it has been proposed to have an important role in cell and tissue function (7). For instance, it can be instrumental in guaranteeing reliable firing in neurons (8); in systems dominated by negative feedback loops, noise can be important for inducing oscillations (9), and it can be important in the generation of intracellular calcium oscillations (10).

In this study we consider the role of cell variability in the function of the hypothalamic–pituitary–adrenal axis, a major physiological regulator of the hormonal stress response. Adrenocortical production and release of corticosteroids [cortisol in humans and corticosterone (CORT) in rodents] is controlled by adrenocorticotropic hormone (ACTH) secretion from pituitary corticotroph cells, which are activated by corticotropin-releasing hormone (CRH) and arginine-vasopressin (AVP) released from hypothalamic neurons (11, 12). CRH and AVP increase the electrical activity of corticotroph cells (13) and increase intracellular calcium concentration ([Ca^2+^]_i_) (14); furthermore, these two neuropeptides have been shown to act in a synergistic manner to enhance the secretion of ACTH (15, 16). The physiological importance and potential impact of corticosteroids on the function of almost all organs leads to the requirement for tightly controlled stress responses; this control is achieved principally through CORT negative feedback both at the level of the pituitary gland and of the hypothalamus (17).

Although previous studies have highlighted marked heterogeneity in the response of corticotrophs to hypothalamic stimuli (18–20), it is unclear whether this population variability derives from stochastic responses of corticotrophs, or whether individual cells respond in a deterministic manner, with differences between subpopulations of corticotrophs. In this study we investigate the heterogeneity in [Ca^2+^]_i_ responses in the corticotroph population: their spontaneous activity, CRH- and AVP-evoked increases in [Ca^2+^]_i_, and their synergy, as well as the effect of CORT exposure on these responses. We show that whereas intrinsic responses of a single cell to repeated challenge are deterministic and reproducible, the corticotroph population displays marked cell-to-cell variability in all of these processes. This property may provide a mechanism that extends the dynamic range and regulates corticotroph output, allowing for appropriate population-level responses and adaptation to different physiological challenges.

## Materials and Methods

### Animals

For all of the experiments, 8- to 10-week-old male Sprague-Dawley rats were kept under standard 12-hour light/12-hour dark conditions, with food and drink supplied *ad libitum*.

All tissue collection was performed between 9:00 and 11:00 am, in accordance with accepted standards of humane animal care, as well as UK Home Office requirements (PPL 60/4349) and University of Edinburgh Ethical Review Committee approval.

### Generation of POMC-GCAMP6s lentivirus

The POMC-GCAMP6s lentiviral reporter construct was generated using a pLenti backbone from Addgene plasmid no. 20946. The coding sequence was replaced with that of the genetically encoded GCAMP6s from Addgene plasmid no. 40753 (21) under the control of the rat minimal POMC promoter (22). This restricts expression to corticotrophs and melanotrophs, with the latter being absent in our preparation, because the intermediate zone is removed.

### Primary cultures of anterior pituitary cells

Anterior pituitary cultures were prepared as previously described (23). Briefly, rats were deeply anesthetized with isoflurane and euthanized by cervical dislocation. After rapidly dissecting the pituitary from a rat, the anterior lobe was isolated with the aid of a scalpel under a dissecting microscope to remove the POMC-expressing melanotrophs of the intermediate zone. The anterior lobes were roughly chopped with a blade, then transferred into a tube containing 2.5 mL of digestion medium [Dulbecco’s modified Eagle medium (DMEM) with 25 mM HEPES, 207 U/mL trypsin, and 36 U/mL DNAse I in DMEM). After 30 to 45 minutes of incubation at 37°C, when most of the tissue was digested, cells were further dissociated mechanically using the tip of a micropipette. The digestion was then blocked using 5 mL of blocking medium (25 mg/mL soybean trypsin inhibitor, 100U/mL aprotinin, and 36 U/mL DNAse I in DMEM). Cells were passed through a 70-µm cell strainer, centrifuged for 10 minutes at 100 × *g* at room temperature, resuspended in growth medium (DMEM, 0.3% bovine serum albumin, insulin–transferrin–sodium selenite, and fibronectin, supplemented with ampicillin, streptomycin, and amphotericin B), and then plated onto 12-mm coverslips.

### Calcium imaging

Anterior pituitary cells were transduced using the POMC-GCaMP6s lentivirus to specifically target expression in corticotrophs. The day after transduction the medium was changed and antibiotics were removed. On the day of the experiment, one coverslip was put under an epifluorescence microscope (Olympus IX81) in a chamber continuously perfused with Ringer’s solution [125 mM NaCl, 2.5 mM KCl, 1.25 mM NaH_2_PO_4_, 12 mM NaHCO_3_, 1 mM MgCl_2_, 2 mM CaCl_2_ (pH 7.3), osmolarity between 300 and 305] bubbled with carbogen. All pharmacological treatments were performed by perfusing the cells with the drug dissolved in Ringer’s solution. Images were collected every second using a 492-nm filter for excitation and a 547/31-nm bandpass filter for emission; fluorescence intensity was calculated using ImageJ, then further analyzed using custom scripts written in R (24). Background subtraction was performed using a region of interest not containing any cell. The area under the curve (AUC), used as a measure of the intensity of the response, was calculated for a 15-minute window from the time the drug arrived in the bath.

### Immunostaining

Cells were fixed for 1 hour with a 4% formaldehyde solution in phosphate-buffered saline (PBS), then washed with PBS. Coverslips were then transferred first in permeabilization buffer (PBS, 0.3% Triton X-100) for 10 minutes, then in blocking buffer (PBS, 0.3% Triton X-100, 3% bovine serum albumin) for 1 hour. Primary antibodies [rabbit anti-ACTH (1:1000), chicken anti–green fluorescent protein (GFP; Abcam ab13970, 1:2000)] were diluted in blocking buffer and cells were left overnight at 4°C on an orbital shaker (Table 1). The following day cells were washed with PBS, then incubated for 1 hour with secondary antibodies [goat anti-chicken immunoglobulin (Ig)Y conjugated with Alexa Fluor 488 (1:1000; Abcam ab150169), goat anti-rabbit conjugated with Alexa Fluor 546 (1:1000; Thermo Scientific A11071] diluted in blocking buffer. After a final wash cells were incubated in a TO-PRO-3 iodide solution for 5 minutes to stain nuclei, then mounted on glass slides using Mowiol 4-88 mounting medium (Calbiochem) containing 1,4-diazabicyclo[2.2.2]octane (Sigma-Aldrich; an antifade agent).

**Table 1.** **Antibodies Used in This Study**

**Peptide/Protein Target**	**Name of Antibody**	**Manufacturer**	**Species Raised in; Monoclonal or Polyclonal**	**Dilution Used**	**RRID**
ACTH	Rabbit anti-ACTH, unconjugated	Abcam ab74976	Rabbit; polyclonal	1:1000	AB_1280736
GFP	Chicken anti-GFP, unconjugated	Abcam ab13970	Chicken; polyclonal	1:3000	AB_300798
Rabbit IgG	F(ab′)_2_ goat anti-rabbit IgG (H+L) secondary antibody, Alexa Fluor 546 conjugate	Thermo Fisher A11071	Goat; polyclonal	1:1000	AB_2534115
Chicken IgY	Goat anti-chicken IgY (H+L) (Alexa Fluor 488)	Abcam ab150169	Goat; polyclonal	1:1000	AB_2636803

Abbreviations: GFP, green fluorescent protein; RRID, research resource identifier.

### Statistical analysis

All statistical analysis was performed using R, using a significance level of *α* = 0.05. Specific statistical tests are indicated in the figure legends. Because the absolute values of the AUC are very variable depending on the levels of expression of the calcium indicator, all statistical calculations have been performed on values normalized to the control group for each experiment. Because most experiments involved repeated exposures of the same cell to different stimuli, AUCs were compared using mixed effects models, using the *nlme* R package. AUC and type of treatment were used as fixed factors; the cell and the experiment were instead used as random factors. A Tukey all-pair comparisons *post hoc* analysis was then performed to compare the effect of different treatments. The percentage of cells activated (or inhibited) by a certain treatment was analyzed using mixed effects logit models followed by a Tukey all-pair comparisons *post hoc* test.

## Results

**Figure 1.**
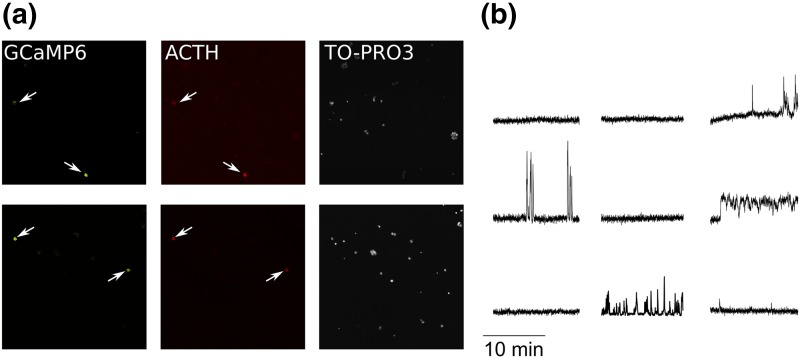
(a) Example of cells transduced using the POMC-GCaMP6s lentivirus. Dispersed pituitary cells were transduced with the virus and fixed 2 days later to be processed for immunocytochemistry. Arrows show cells positive for GCaMP6 and ACTH. Nuclei are stained using TO-PRO-3 iodide. (b) Representative examples of spontaneous [Ca^2+^]_i_ activity in nine corticotrophs, showing large heterogeneity in the population. Traces are shown as normalized between 0 and 1.

A lentiviral vector expressing the genetically encoded calcium indicator GCaMP6s (21) under the control of a minimal POMC promoter (22) was used to specifically target corticotrophs in rat anterior pituitary primary cultures. The vector was able to transduce ACTH-immunoreactive cells with >95% specificity [Fig. 1(a)], consistent with a similar viral construct expressing enhanced yellow fluorescent protein (23).

Spontaneous calcium activity was generally low in corticotrophs [Fig. 1(b)], with only ∼30% showing any spontaneous calcium transients during the recording period. These events were highly variable in terms of amplitude, duration, and kinetics, and they included both high frequency spiking as well as sustained elevations that lasted for several minutes.

**Figure 2.**
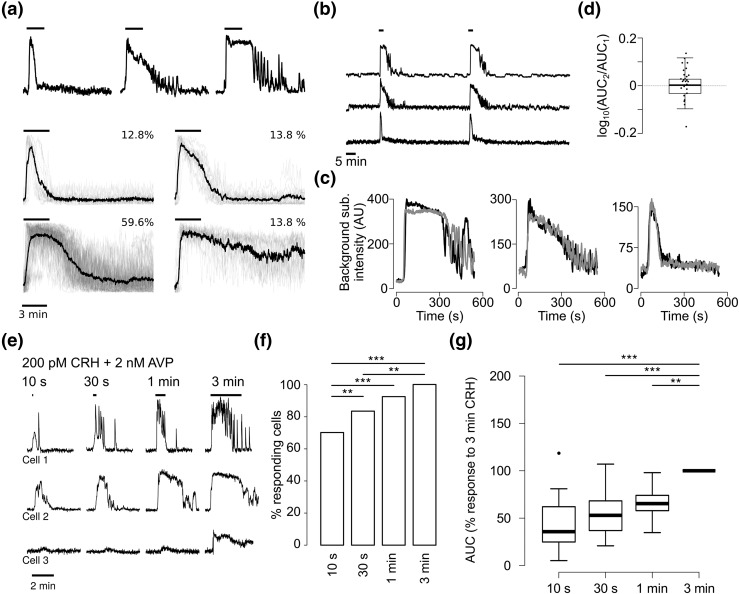
(a) Top: Example of three corticotrophs treated simultaneously with 200 pM CRH and 2 nM AVP for 3 minutes (indicated by black bar), showing large heterogeneity in the calcium response to the stimulus. Bottom: Responses to 3-minute CRH plus AVP stimuli in the corticotroph population, classified depending on their [Ca^2+^]_i_ response (n = 94 cells from 10 experiments; black line shows mean response, gray lines show individual traces; black bars indicate period of exposure to CRH plus AVP). In all of the panels, traces are shown as normalized between 0 and 1, and the *y*-axis is omitted for visual clarity. (b) Responses of the three corticotrophs in (a) to two successive applications of CRH plus AVP. Black bars indicate time of exposure to CRH plus AVP. (c) Superposition of extracts of the two traces shown in (b), showing deterministic [Ca^2+^]_i_ changes in corticotrophs after secretagogue stimulation. Black line shows response to the first challenge (starting at 15 minutes); gray line shows response to the second challenge (starting at 45 minutes). (d) Box plot showing the log-transformed ratio of the AUC of the Ca^2+^ response to two exposures to CRH plus AVP. The log ratio is not different from 0 (n = 26 cells from three experiments, *P* = 0.86, one-sample *t* test). (e) Examples of three corticotrophs stimulated with 200 pM CRH plus 2 nM AVP for increasing amounts of time, showing heterogeneity in the minimum amount of exposure required to have a response. Black bars indicate time of exposure to CRH plus AVP. (f) Cumulative percentage of cells responding to the protocol shown in (e). **P < 0.001, ***P < 0.0001, logit model, Tukey *post hoc* test. (g) Box plot of AUC of the cells, expressed as percentage of the longest treatment of the experiment shown in (e) (n = 32 cells from four experiments). **P < 0.01, ***P < 0.001, mixed effects model.

When challenged for 3 minutes with 200 pM CRH plus 2 nM AVP, corticotrophs responded with a rapid increase in [Ca^2+^]_i_, followed by a variable return to baseline [Fig. 2(a), top]. In 12 out of 94 cells the response ended before the end of the stimulus; in 13 cells the [Ca^2+^]_i_ levels started decreasing during the stimulus, but returned to baseline only after its end; in 56 cells the response was sustained during the stimulus, and the response rapidly decreased to baseline levels following the end of the stimulus. The remaining 13 cells showed a sustained response even after the stimulus ended [Fig. 2(a), bottom].

To test whether this heterogeneity of responses derived from variability at the level of the population (each cell responding in a different way), or whether the responses were stochastic (each cell responding each time stochastically in one of several possible ways), we challenged cells twice with the same CRH plus AVP stimulus. Responses in this case were deterministic and highly reproducible [Fig. 2(b–d)], even when the stimulus was repeated up to five times. Heterogeneity was also evident in the sensitivity of cells to the length of exposure to CRH/AVP [Fig. 2(e–g)]. We observed a stimulus length-dependent increase in the percentage of cells that responded to CRH/AVP; whereas all 32 cells analyzed (n = 4 experiments) responded to a 3-minute exposure to the secretagogues, briefer exposures did not always elicit calcium responses, with nine out of 32 cells not responding to 10-second exposure, five not responding to a 30-second exposure, and two not responding to a 1-minute exposure [Fig. 2(f)].

**Figure 3.**
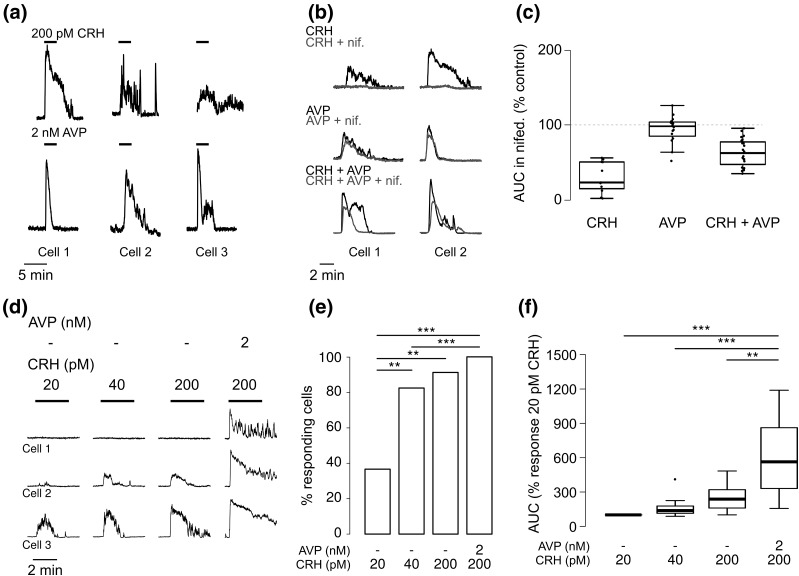
(a) Examples of responses of three different cells to 200 pM CRH (top) or 2 nM AVP (bottom), showing heterogeneous responses in the cell population. (b) Effect of nifedipine (nif.) on calcium responses to 200 pM CRH, 2 nM AVP, or their combination. Gray lines show response in nifedipine (nifed.). (c) Box plot showing AUC of the calcium responses to the protocol shown in (b), expressed as percentage of the control exposure. Exposure to nifedipine significantly decreases AUC in cells treated with CRH (n = 16, *P* < 0.05 mixed effects model) or CRH plus AVP (n = 23, *P* < 0.001), but not AVP (n = 11, *P* = 0.99). (d) Examples of the calcium responses of three corticotrophs stimulated with different doses of CRH or CRH plus AVP for 3 minutes, showing heterogeneity in the minimum dose required to have a response. (e) Cumulative percentage of cells responding to the protocol shown in (d). **P < 0.001, ***P < 0.0001, logit model, Tukey *post hoc* test. (f) Box plot of AUC of the cells, expressed as percentage of the treatment with 200 pM CRH and 2 nM AVP (n = 11 cells from three experiments). **P < 0.001, ***P < 0.0001, mixed effects model.

Having noted marked heterogeneity in the responses to a combined CRH plus AVP stimulus, we investigated whether cell responses to a single secretagogue were similarly variable. Indeed, both responses to 200 pM CRH and to 2 nM AVP were found to be heterogeneous [Fig. 3(a)] and, as previously reported (14), dependent on different molecular pathways; CRH responses were abolished by treatment with the L-type calcium channel blocker nifedipine, indicating that calcium influx from the extracellular medium drives these responses. Alternatively, nifedipine did not significantly affect AVP responses and only partially, albeit significantly, reduced responses to the combined treatment [Fig. 3(b) and 3(c)]. Further variability was observed in the minimal CRH concentration eliciting a response: whereas all of the 11 cells analyzed (n = 3 experiments) responded to the 200 pM CRH plus 20 nM AVP stimulus, only 10 responded to 200 pM CRH, five to 40 pM CRH, and four to the lowest tested concentration of 20 pM CRH [Fig. 3(d) and 3(e)]. This was accompanied by a dose-dependent increase in the AUC of the [Ca^2+^]_i_ response to the secretagogues [Fig. 3(f)].

**Figure 4.**
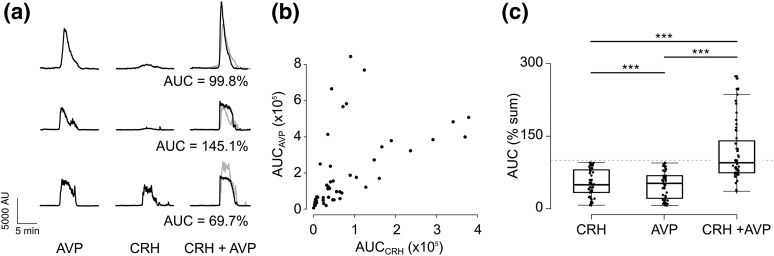
(a) Fifteen-minute excerpts from recordings of three corticotrophs treated sequentially with 2 nM AVP, 200 pM CRH, and the combination of the two, showing different levels of synergistic Ca^2+^ responses. The proportion of the AUC of the CRH plus AVP treatment and the sum of the single treatments, used as a measure of synergy, are reported under each trace. Gray lines show the theoretical sum of the responses to CRH and AVP. (b) AUC of calcium responses following subsequent exposure to CRH and AVP for the cells shown in (a). (c) Box plot showing the AUC of the calcium responses from cells treated as in (a), expressed as percentage of the sum of CRH and AVP responses (n = 51 cells from 11 experiments). ***P < 0.001, mixed effects model. The combined treatment (CRH plus AVP) was not statistically different from the sum of CRH and AVP (*P* = 0.91, mixed effects model).

Synergy between CRH and AVP on ACTH secretion has been widely described. To determine whether it occurs also at the level of [Ca^2+^]_i_ responses to hypothalamic secretagogues, cells were treated with the two hormones separately before adding them together. To counteract any effect of the order of exposure to the single treatments, their sequence was randomized [*i.e.*, CRH, AVP, combination, n = 28 or AVP, CRH, combination, n = 23 from 11 experiments; Fig. 4(a)]. Responses to CRH and AVP did not appear strongly correlated in magnitude; at the doses used in this study, AVP responses were always stronger or equal in magnitude to the responses to CRH. In 40% of the recorded cells, CRH responses were attenuated, and <50% in magnitude, when compared with the response to AVP in the same cell [Fig. 4(b)]. The AUC of the combined treatment was then compared with the sum of the areas of the single treatments. Although the AUC of the combined treatment was not statistically different from the sum of the single treatments when considering the population [Fig. 4(c)], in 20 of 51 cells (39.2%) the AUC of the combined treatment was greater than the sum of the single treatments.

**Figure 5.**
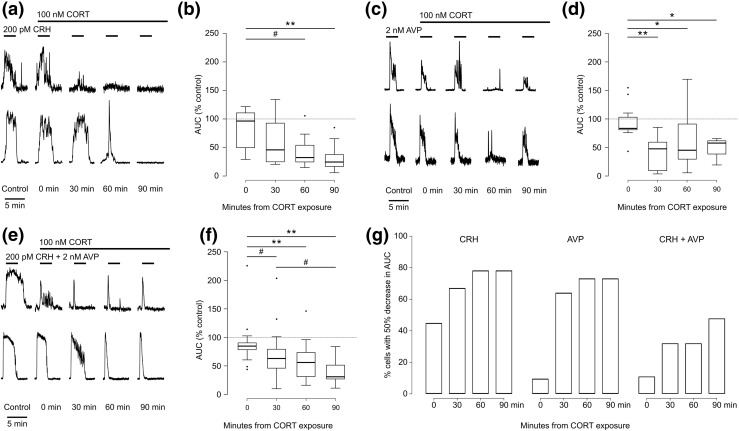
(a) Example of calcium responses from two corticotrophs treated with 200 pM CRH for 3 minutes and then repeatedly challenged with the same stimulus at 30-minute intervals in the presence of 100 nM corticosterone. The lines above the trace show the timing of the exposure. (b) Box plot of AUC for the responses from the experiment shown in (a), expressed as percentage of control exposure (n = 9 cells from three experiments). #*P* < 0.05, **P < 0.001. All groups except the “0 minutes” group are different from control (*P* < 0.01, mixed effects model). (c and d) As in (a) and (b), but for exposure to 2 nM AVP (n = 9 cells from four experiments). *P < 0.01, **P < 0.001, mixed effects model. All groups but the “0 minutes” group are different from control (*P* < 0.01, mixed effects model). (e and f) As in (a) and (b), but for exposure to 200 pM CRH plus 2 nM AVP (n = 12 cells). #*P* < 0.05, **P < 0.001, mixed effects model. All groups except the “0 minutes” group are different from control (*P* < 0.001, mixed effects model). (g) Bar plot showing the cumulative percentage of cells with a 50% decrease in the AUC over time. The 30- and 60-minute groups are different from 0 (*P* < 0.05), and the 90-minute group is different from 0 (*P* < 0.001, logit model, Tukey *post hoc* test). Treatment with CRH and treatment with AVP are significantly different from treatment with CRH plus AVP (*P* = 0.03, logit model).

Finally, we assessed the effect of CORT treatment on the [Ca^2+^]_i_ responses to CRH, AVP, or their combination. Cells were first treated with the secretagogue(s) alone; this was then repeated a further four times, every 30 minutes, in the presence of 100 nM CORT [Fig. 5(a), 5(c), and 5(e)]. Heterogeneity in CORT effects was again apparent, ranging from immediate reduction of the responses to reduction in their amplitude or their duration. Analysis of the entire population of cells showed that the reduction in calcium responses was statistically significant between 30 and 90 minutes after CORT exposure [Fig. 5(b), 5(d), and 5(f)]. Overall, there was a statistically significant time-dependent increase in the percentage of cells showing >50% reduction in their [Ca^2+^]_i_ response to secretagogues after exposure to CORT [Fig. 5(g)]. The dynamics of inhibition from CORT exposure differed among the three treatments: specifically, an immediate decrease of the response was seen in four of the nine cells treated with CRH [Fig. 5(g), left], whereas at the same time CORT was only able to inhibit one of nine cells treated with AVP or two of 12 cells treated with CRH plus AVP [Fig. 5(g), middle and right]. Thirty minutes after CORT exposure, more than half of the cells treated with either CRH or AVP were inhibited, and this inhibition lasted to a similar level for up to 90 minutes of exposure to CORT both in terms of AUC [Fig. 5(b) and 5(d)] and of percentage of cells inhibited [Fig. 5(g)]. In the cells treated with CRH and AVP, however, a statistically significantly smaller number of cells were inhibited at all times [Fig. 5(g)], and the maximum inhibition of AUC was only seen 90 minutes after CORT exposure.

## Discussion

The regulation of pituitary gland function by hypothalamic factors and its modulation by target organ feedback are essential for maintaining optimal output of a number of neuroendocrine axes. Different degrees of functional heterogeneity in the response to secretagogues have been reported for a number of pituitary cell populations, including somatotrophs (25), gonadotrophs (26), lactotrophs (27), and corticotrophs (18), suggesting this as an efficient evolutionary mechanism to enhance hormonal responses from the pituitary gland. In this study, we have used changes in corticotroph [Ca^2+^]_i_, which have been related to changes in membrane capacitance, indicative of ACTH granule fusion and thus secretion (28), to determine cellular heterogeneity in hypothalamic stimulation and feedback regulation. We report a marked heterogeneity in the responses to secretagogues in rat corticotrophs, which is apparent at multiple levels when analyzing intracellular calcium responses. Our data suggest that a deterministic mechanism underlies the regulation of corticotroph cells to generate appropriate physiological responses to stressors.

In our study, heterogeneity of corticotroph cell activity was already apparent when spontaneous calcium activity was monitored; corticotrophs from male rats showed a variety of behaviors, ranging from being “silent” (the majority), to having high spontaneous activity. Heterogeneity in corticotroph spontaneous electrical activity has been previously reported in both male and female mice (13, 23, 29). Interestingly, an earlier study in female rat corticotrophs identified by immunohistochemistry (30) reported spontaneous [Ca^2+^]_i_ activity in 65% of cells, possibly indicating the presence of sex differences in the activity of these cells.

When cells were challenged with hypothalamic secretagogues, further heterogeneity in [Ca^2+^]_i_ activity was apparent in the pattern, duration, and dose-sensitivity of the response. Strikingly, marked heterogeneity was seen between cells in terms of the intensity and duration of the response, and this was deterministic for each cell. Heterogeneity in calcium responses of corticotrophs to high concentrations of AVP has been previously noted as a result of the activation of different ionic currents in these cells (18). Variability in the basal and CRH-stimulated secretion of ACTH, as measured by reverse hemolytic plaque assay, has also been previously described in corticotrophs (19), suggesting that this heterogeneity is relevant for endocrine signaling. Similarly, variable activation of c-Fos after a CRH stimulus has been previously noted in adult male rat corticotrophs (20). Whether this variability of responses implies the existence of subpopulations of corticotrophs is unclear; electron microscopic observations have shown morphological differences in the corticotroph population, suggesting the existence of four different subtypes of corticotrophs (31), although functional correlates of these anatomical differences have not been described. At the molecular level, three subpopulations of corticotrophs have been identified in sheep fetuses in relationship to their gene expression signature after stress (32). It is not at present known whether these represent real subpopulations of cells of different origin. Rather than thinking of static subpopulations of cells, it is reasonable to assume that the specific “fingerprint” of channel expression in each cell, and variability in their amount, will determine its specific response, and that this may change over time. Mathematical modeling of experimental data suggests that cell-to-cell variations in even a small number of currents are sufficient to generate variation in spontaneous and secretagogue-induced electrical activity in corticotrophs (33); similarly in lactotrophs, variation in calcium responses has been proposed to depend mainly on variation in calcium extrusion from membrane Ca^2+^ adenosine triphosphatase, and on cell-to-cell differences in endoplasmic reticulum calcium levels (27). Previous studies have shown marked anatomical and functional plasticity in corticotrophs during different physiological states (34), suggesting that corticotrophs may be a single population that can exist in a large spectrum of states, with individual cells switching state following stimuli of different type or intensity. Similarly, we found that increasing doses of CRH had the effect of both increasing the magnitude of [Ca^2+^]_i_ responses and of recruiting more cells to respond. A recent study showed a similar dose-dependent increase in the number of cells showing calcium responses in response to CRH treatment in mice (29). Interestingly, the same study reports inhibition of calcium responses at CRH concentrations higher than those used in our study. Dose-dependent discrete activation of subpopulations of cells has been described in terms of hormone secretion from corticotrophs (35), as well as from gonadotrophs (36) and somatotrophs (37), and it has also been noted in nonpituitary systems (38). This combination of “analogue” (size of response) and “digital” (percentage of responding cells) could permit an increase in the dynamic range of cellular responses by encoding stimuli both at the single-cell and at the population level. Furthermore, this heterogeneity in responses to secretagogues may allow linear responses to a large range of stimuli, in a similar fashion to that of vasopressin neurons, where it has been described as enabling a linear output from a nonlinear population of cells (39).

Interestingly, there was no constant relationship between the responses of corticotrophs to the two hypothalamic stimuli. Elevation of intracellular calcium following a CRH stimulus is predominantly nifedipine-sensitive and dependent on calcium influx via activation of L-type calcium channels (40, 41). In contrast, increase in intracellular calcium following AVP stimulation is largely dependent on release from intracellular calcium stores (42). In our experiments, nifedipine inhibited responses to a combination of CRH and AVP in a variable, cell-dependent way, suggesting that the relative nifedipine-sensitive and -insensitive components of the responses were heterogeneous in the population. This was further confirmed when treating the same cells in sequence with the two independent stimuli; responses were observed ranging from strong responses to both CRH and AVP to attenuated responses to either factor.

An important characteristic of ACTH secretion from corticotrophs is that the two hypothalamic secretagogues CRH and AVP act in a synergistic manner; this has been previously described in primary rat anterior pituitary cultures (15, 43) as well as *in vivo* (16). This synergy is also reflected at the level of second messenger production, where potentiation of cyclic adenosine monophosphate production in the presence of both hormones is mediated by the activation of adenylate cyclase and inhibition of phosphodiesterases (43). Furthermore, activation of protein kinase C has also been implicated in the generation of synergistic responses, through inhibition of TREK currents (44). Our data show that, although at the population level no significant synergistic calcium responses are seen, at the level of single cells, ∼40% of cells show synergistic [Ca^2+^]_i_ responses to CRH and AVP; this further supports the notion that intracellular pathways are heterogeneous in the corticotroph population.

Finally, we have analyzed the effects of CORT on the [Ca^2+^]_i_ responses of corticotrophs. The effects of CORT on the activity of corticotrophs are well known; pretreatment with CORT for 90 minutes has been shown to decrease firing rate and event duration measured from single corticotrophs (45). Treatment with CORT has been shown to gradually reduce CRH- or AVP-induced ACTH secretion from perfused pituitary cells in a time frame similar to the population data from our study (46, 47). Our results show a progressive recruitment of cells for the response to glucocorticoids, during the course of 90 minutes, which could be instrumental for the generation of gradual responses to CORT. This is in agreement with data showing a decrease in the number of plaque-forming corticotrophs in a reverse hemolytic plaque assay (35), although in that study cells were pretreated with CORT for several hours. Furthermore, the effects of CORT were less pronounced when cells were treated with both CRH and AVP compared with when a single secretagogue was used. This is in line with recent electrophysiological reports showing that treatment with CRH plus AVP is able to overcome CORT-induced hyperpolarization of corticotrophs, whereas CRH alone is not (45).

In conclusion, we have shown that a high degree of heterogeneity exists in the corticotroph population at the level of [Ca^2+^]_i_ responses to hypothalamic secretagogues, synergistic responses to CRH and AVP, as well as CORT inhibition. This highlights the importance of performing single-cell analysis when considering regulatory and feedback mechanisms controlling pituitary gland output, because integration of variable cell responses may be an important component underlying the function of endocrine systems.
